# Bone mineralisation and glucose metabolism

**DOI:** 10.1016/j.coemr.2023.100446

**Published:** 2023-04

**Authors:** Fiona L. Roberts, Vicky E. MacRae

**Affiliations:** 1 Center for Clinical Research, Lund University, Malmö, Sweden; 2 The Roslin Institute and Royal (Dick) School of Veterinary Studies, University of Edinburgh, Easter Bush, Midlothian, UK

**Keywords:** Bone, Glucose, Diabetes, Mineralisation

## Abstract

Recent advancements in the bone biology field have identified a novel bone-metabolism axis. In this review, we highlight several novel studies that further our knowledge of new endocrine functions of bone; explore remaining unanswered questions; and discuss translational challenges in this complex era of bone biology research.

## Introduction

Bone is an active tissue that undergoes constant remodelling to meet the mechanical demands of mobile organisms. This continual remodelling of bone material is mediated by the bone-forming cells, known as osteoblasts, and the bone-resorbing cells known as osteoclasts. Beyond this, bone tissue includes additional cell types such as bone lining cells and osteocytes, posited to act as mechanosensors and overall orchestrators of the bone remodelling process [1]. Both local and systemic factors influence bone formation and resorption, altering this dynamic process. This is highly complex and involves orchestrated interplay of bone formation and pro-mineralisation factors, together with bone resorption and anti-mineralisation regulators (Table 1). This process is energetically demanding, requiring synthesis and degradation of multiple proteins and organic matrix, with its subsequent mineralisation to retain the structural and mechanical capacity of formed bone.

**Table 1:** Summary of selected mineralisation regulators.

Mineralisation factor	Role in Bone
Ectonucleotide Pyrophosphatase/Phosphodiesterase 1 (ENPP1)	Inhibits mineralisation by generating pyrophosphate
Ankylosis protein (ANK)	Inhibits mineralisation by transporting ATP and PPi to the extracelluar space
Tissue-nonspecific alkaline phosphatase (TNAP)	Drives mineralisation by generating phosphate
type III NaP_i_transporters (Pit1/2)	Drives mineralisation by transporting phosphate to the intracellular space
Phosphoethanolamine/Phosphocholine Phosphatase 1 (PHOSPHO1)	Initiates mineralisation by generating phosphate
Osteopontin OPN	Inhibits mineralisation by binding hydroxyapatite
Matrix Gla Protein MGP	Inhibits mineralisation by binding hydroxyapatite

Recent advancements in the bone biology field have identified a bone-metabolism axis: indeed bone-specific hormones (e.g. osteocalcin), bone-related hormones (e.g. adiponectin from bone marrow adipose tissue) and metabolism-influencing roles of bone mineralisation regulators have been described in the last decade [2]. Given that there are links between bone disorders and metabolism such as diabetic bone disease, evidenced at the clinical and basic biology level, it is pertinent to further investigate the metabolic links between bone mineralisation and glucose metabolism. This may serve to identify novel targets to improve both bone health and conditions of dysregulated glucose metabolism.

## Physiological roles of selected mineralisation regulators in relation to bone and glucose metabolism

Recent work has illuminated the metabolic plasticity and function of glucose metabolism in osteoclast and osteoblast physiological function including differentiation and mineralisation regulation functions. This work has highlighted the intersections between various bone-associated growth/transcription factors, which mediate the function of osteoblasts and osteoclast cells, along with systemic glucose metabolism. An increased fundamental understanding of glucose metabolism pathways and their role in critical actions of bone cells will identify novel therapeutic options for both bone and glucose homeostasis, and areas where this (patho)physiology overlap.

Initial observations of mineralisation regulation and links with systemic homeostasis involved the identification of bioactive osteocalcin (an osteoblast activity marker), secreted from the osteoblast and reportedly acting systemically to increase pancreatic β-cell proliferation and insulin secretion [3]. In addition, systemic insulin levels correlate with bone remodelling markers, suggesting a link between osteoblast activity and glucose homeostasis [4]. The endocrine function of bone cells is further highlighted through the actions of Fibroblast growth factor 23 (FGF23), a phosphaturic osteocyte-derived hormone that participates in phosphate and vitamin D metabolism [5].

### Ectonucleotide pyrophosphatase phosphodiesterase 1

Evidence suggests that inhibitors of mineralisation, both in bone and soft tissue, also regulate glucose metabolism. The enzyme ectonucleotide pyrophosphatase phosphodiesterase 1 (ENPP1) is recognised as a pathogenic factor predisposing to insulin resistance in humans by allosterically modulating the insulin receptor and negatively impacting insulin pathways [6]. Specific mutations in *ENPP1* are associated with increased insulin resistance in humans and show diversity between ethnic groups [7]. In mouse models, ablation of *Enpp1* results in profound resistance to obesity and insulin resistance following a high-fat diet challenge [8]. Furthermore, an osteoblast-specific *Enpp1* knockout mouse model recently revealed an impaired glucose homeostasis and insulin tolerance following high-fat diet feeding, coupled with increased mineralisation capacity of osteoblasts and a notable sexually dimorphic bone phenotype with increased bone volume in females [6]. Together these data indicate that ENPP1 plays a crucial part in metabolic regulation, although continued work is required to further delineate the cell-specific contributions and molecular pathways underpinning these findings. Furthermore, ENPP2 (autotaxin), an adipocyte-derived lysophospholipase, has been implicated in obesity and insulin resistance in animals and is identified as a novel factor mediating osteoclast function and bone loss, further highlighting the role of NPP family members in the mediation of glucose metabolism and bone health [9].

### Phosphatase orphan 1

The bone-specific phosphatase, phosphatase, orphan 1 (PHOSPHO1) plays a central role in the regulation of bone mineralisation [10] and has recently been identified as a candidate responsible for non-osteocalcin mediated osteoblast regulation of glucose metabolism. This is evidenced in human studies demonstrating that PHOSPHO1 is a key regulator of energy metabolism, revealing that PHOSPHO1 methylation sites can be used as a biomarker for the early detection of T2DM [11]. It is suggested that ablation of PHOSPHO1 may translate into improved metabolic outcomes such as reduced metabolic disease risk and improved glucose regulation. Indeed, *Phospho1* deficiency in mice results in a reduced blood glucose concentration, enhanced insulin sensitivity and improved glucose tolerance protecting high-fat diet-induced obesity [11]. Parallel studies have further revealed a novel role for PHOSPHO1 as a negative regulator of brown adipose tissue thermogenesis, and have identified the inhibition of PHOSPHO1 or enhancement of the PHOSPHO1 substrate phosphocholine as innovative approaches to manage the metabolic syndrome [12].

### Tissue non-specific alkaline phosphatase

Tissue non-specific alkaline phosphatase (*TNAP)* is principally recognised as a regulator of hydroxyapatite formation, important for mineralisation-competent tissues including bone, teeth and the vasculature. In this context, *TNAP* is responsible for hydrolysing PPi to generate Pi for matrix vesicle-mediated mineral formation [13], therefore acting as a pro-mineralisation regulator. In the last decade, there has been an accumulation of evidence that *TNAP* also functions in adipose tissue, mediating cell lineage commitment and adipose tissue functionality. It has been demonstrated that TNAP is associated with mesenchymal progenitor differentiation towards mineralising osteoblast and chondrocytes. There is additional evidence that TNAP activity in mesenchymal progenitor cells induces adipocyte differentiation, revealing a new physiological role for this alkaline phosphatase which may have implications for glucose homeostasis and energy metabolism [14]. It is important to note that there are data which contradict this reported function, instead suggesting that TNAP inhibits mesenchymal progenitor differentiation towards adipocytes [15]. Utilising cell models of adipocytes, the expression of TNAP has been shown to increase in parallel will cellular differentiation, with TNAP expression observed in subsets of beige adipocyte progenitors [16]. These studies further indicate that specific knockdown of TNAP mRNA levels results in the inhibition of triglyceride accumulation [16]. The precise molecular machinery mediating the effect of TNAP on adipocyte lipid accumulation and differentiation remains incompletely understood, although it has been suggested that this is regulated through decreased purinergic receptor activation [17]. Interesting observations have also been reported in the field of brown and beige adipocyte biology in relation to TNAP function. TNAP has been implicated in the regulation of brown adipose tissue thermogenic function. Notably, the expression of TNAP is increased in adipocytes that have been cold-exposed, with localisation of TNAP within the inner mitochondrial membrane observed. The role of TNAP in brown adipose tissue thermogenesis is suggested to be related to the mechanism of the futile creatine cycle, whereby energy is dissipated by the generation of Pi as opposed to ATP from the TNAP-mediated hydrolytic breakdown of phosphocreatine in adipose cells [18,19]. Collectively, these data indicate that TNAP has a crucial role in the regulation of mineralisation, with secondary emerging roles in the regulation of adipose tissue physiology. As such, the function of TNAP may play a key role in the regulation of adipocyte energy balance and may be a mediator of obesity and metabolic syndrome. There is therefore a need to provide a deeper explanation of the specific mechanism of action through which such function is achieved, necessitating continued and further assessment of TNAP in the context of extra-skeletal tissues in relation to the regulation of obesity, metabolic syndrome and glucose homeostasis.

### Osteopontin

Osteopontin is a protein with multiple functions which is expressed in a variety of cell types including adipocytes and bone tissue, which represents the major site of production. Its role in bone is to regulate mineralisation is achieved by binding to hydroxyapatite, which potently inhibits the growth of hydroxyapatite crystals and inhibits the mineralisation process [20]. Daniele and colleagues recently showed, through an investigation of patients diagnosed with T2DM, that osteopontin levels where increased by two fold compared to normal glucose-tolerant subjects [21]. This study indicated that, as glucose tolerance worsens in humans, the levels of circulating osteopontin increase. Further investigation demonstrated increased osteopontin levels in association with both type 1 and type 2 diabetes [21]. Given that osteopontin is known to regulate bone mineralisation, and that recent findings indicate its association with T2DM, it is plausible that osteopontin may be a novel mediator regulating both skeletal and glucose metabolic homeostasis. Additional studies are therefore required to determine the relationship of bone marker levels with glucose metabolism, and to determine whether there are causative molecular mechanisms underpinning this association. Despite this information being scarce, such studies further point towards complex organ crosstalk mechanisms whereby bone, and its associated regulators, are increasingly implicated in the regulation of whole-body metabolism and glucose homeostasis. This is of particular interest in the case of at-risk populations who may experience both metabolic and bone pathologies, such as in the case of type 2 diabetes associated diabetic fracture, and in elderly populations whereby there is increased risk of type 2 diabetes development and osteoporosis concomitantly [22].

## Bone-adipose axis

Given that body weight impacts both bone health (in terms of bone turnover rate and bone mineral density) and the risk of developing T2D, it is also important to consider mechanisms related to the fat-bone axis. Adiponectin is an adipocyte-derived hormone that is implicated in the regulation of bone function, and it has been reported that adiponectin acts directly on bone cells, increasing the proliferation of osteoblasts and the inhibition of osteoclastogenesis *in vitro*, posited to facilitate a net increase in bone mass [23]. It has been further shown that adiponectin signals in the brain, mediated by forkhead box protein 01 (Fox01), resulting in sympathetic nervous system inhibition which results in increased formation of bone and increased bone mass [24]. Interestingly, Kajimura and colleagues also demonstrate that adiponectin acts through local signalling mechanisms within the osteoblast which prevent osteoblastogenesis and resulting in a reduction of bone formation and bone mass [24].

An additional adipose–bone axis player that has gained traction in recent years is leptin. Initially identified as a key regulator of energy homeostasis and energy expenditure, there is increasing evidence of its role in bone metabolism. It has been demonstrated that leptin acts through both direct and indirect mechanisms to mediate bone metabolism. In bone, receptors for leptin have been found in adult primary osteoblasts and chondrocytes, suggesting a potential direct effect of leptin on these target tissues. Furthermore, leptin also activates fibroblast growth factor 23 (FGF23) which may mediate its regulation of bone growth [25]. Peripheral mechanisms may also explain the role of leptin in the inhibition of bone formation, mediated by signalling in the ventromedial hypothalamus. It is postulated that this signalling may activate the local noradrenergic signals in the osteoblast to influence bone mass through a hypothalamic relay [26]. As such, there is potential for leptin to be used as a pharmacological tool to address metabolic bone disease.

Whilst the exact function of adiponectin and leptin regarding bone health has yet to be fully elucidated, it is evident that nutrition-related peptides play a role in mediating bone mass: this area requires continued investigative efforts.

## Mineralisation and glucose regulation in metabolic disease

The interplay of bone mineralisation regulation and systemic glucose metabolism is illuminated in pathophysiological conditions. For example, it is well known that type 2 diabetes mellitus (T2DM) is associated with normal-to-high bone mineral density (BMD) and concomitant significantly increased fracture risk: this can be termed as diabetic bone disease [27]. Dysregulated glucose metabolism due to failure of pancreatic β-cells to secrete sufficient insulin is the main culprit of T2DM and results in patients experiencing hyper- and hypoglycaemic events, accompanied by obesity. In both obesity and T2DM, there are notable changes to the levels of osteokines and bone mineralisation regulators, including osteocalcin, osteoprotegerin and sclerostin [28]: this is of considerable interest given that metabolic syndrome is associated with diminished skeletal health including decreased BMD [29]. It is also suggested that high glucose concentrations cause an alteration in purinergic signalling, a process also mediated by bone mineralisation regulators such as ENPP1, which results in impaired ATP signalling [30]. Impaired ATP signalling is strongly associated with both bone mineralisation and metabolic disorders. Indeed, it has been suggested that the P2X7 receptor-Pannexin1 interaction within osteocyte mechano-transduction is a key player in ATP release in bones and that high glucose conditions result in impaired load-induced ATP release from osteoblasts and posited alterations to bone health maintenance [31]. Together these data indicate a putative link between bone regulators and metabolism centred around ATP pathways [32].

Chronic kidney disease (CKD) is a common concomitant disease associated with T2DM. Here, patients experience CKD-mineral bone disorder which underpins the development of diabetic bone disease and increased bone fragility and fracture incidence. In CKD, a disordered calcium-vitamin D-PTH axis exists which promotes the loss of bone mass and mineral density [33]. Patients who have improved glucose homeostasis present with reduced levels of calcium excretion via the urine, preventing further calcium leaching from the minerals of bones, thus normalising BMD [33]. The regulation of mineral metabolism hinges on parathyroid hormone (PTH), FGF23, calcitriol and other essential players which mediate bone mineralisation: for example there is emerging evidence of cross-talk between adipose tissue and calcium/phosphorous homeostasis suggesting an interplay between metabolic disorders associated with adipose tissue and the PTH-axis [34]. Clinical evidence demonstrates a decrease in adipose tissue following alterations to the mineral profile, whilst those patients with fat metabolism pathologies demonstrate altered calciotriopic hormone levels [34]. Identifying the interlinking players in altered fat metabolism and CKD-related mineral disturbances is key to determining how these two homeostatic systems influence each other: this may identify novel therapeutic targets to tackle CKD incidence amongst T2DM diagnosed individuals who typically present with notably enhanced adipose tissue, disrupted adipose health and dysregulated glucose metabolism.

Recent studies have further sought to elucidate the role of caloric restriction on glucose metabolism and associations with bone health. Of interest, there are notable sexual dimorphisms at the level of adiposity, insulin sensitivity and circulating metabolic-regulating hormones as a product of reduced dietary protein intake in mouse models of multiple strains. Overall, metabolic health is improved by a reduction in dietary protein, and this is contingent upon genetic background and sex, as evidenced through a multi-omics approach [35]. Particularly, this study identified that the metabolic and molecular phenotypes, including circulating Fibroblast growth factor 21 (FGF21) levels and FGF21 expression in various metabolic tissues, are highly variable between strains and sex [35]. Given that bone loss, and dysregulation of glucose metabolism, is mediated by FGF21 [36], such evidence further supports the synergistic regulation of bone and metabolic health, reinforcing the need for careful appraisal of results dependent upon genetic background and sex, and highlighting the requirement for personalised therapeutic interventions.

## Mitochondrial health

To elucidate the implications of T2D in bone cells, high glucose has been used to mimic the disease *in vitro*. Studies have shown that high glucose inhibits matrix mineralisation in MC3T3-E1 osteoblast-like cells [37], with both mitochondrial respiration and glycolysis maintaining energy demands [38]. Identifying the key pathways that mediated mitochondrial functions in bone cells will therefore give a critical bioenergetic map of metabolic-related bone disease and pathways and bone-related axis mediating systemic glucose metabolism.

There is increasing evidence demonstrating the importance of mitochondrial function particularly in osteoclasts and osteocytes. During the osteogenic differentiation process, there is a significant increase in both mitochondrial biogenesis and mitochondrial activity [39]. Furthermore, recent evidence indicates that mitophagy (the selective autophagy of mitochondria) may play a vital role in regulating the proliferation, differentiation and function of osteoblasts and osteoclasts. The process of mitophagy facilitates the maintenance of the appropriate number of functional mitochondria to match the energy demand of the cells [40]. If mitophagy is disturbed or dysregulated, an accumulation of damaged mitochondria occurs which can induce either osteoblastic apoptosis or osteoclastogenesis in bone [41]. Several signalling mechanisms, including PINK1/Parkin, SIRT1, FOXO3, Beclin-1, p62 and mTOR pathways, have been implicated in the regulation of mitophagy in these cells [40]. However, the role of mitophagy on bone cells remains understudied, with further investigation essential to fully elucidate the involvement of mitophagy within the bone-metabolism axis.

Interestingly, recent studies have also identified mitochondrial dynamics as a potential therapeutic target for clinical intervention against soft tissue mineralisation, through an osteocalcin/Wnt/B-catenin signalling pathway [42]. Osteocalcin knockout vascular smooth muscle cells (VSMCs) show higher basal glycolysis and maximal respiration rates compared with wild-type VSMCs, which is indicative of these cells having greater energy demand, and increased ATP-linked respiration. This in turn may inhibit mineralisation in an ENPP1-dependent manner, whereby ENPP1 converts ATP to pyrophosphate, a potent calcification inhibitor [6]. Further studies employing ENPP1-deficient mouse models could be employed to investigate the role of pyrophosphate biogenesis more fully as a key component of mitochondrial contributions to the mineralisation process downstream of this newly elucidated osteocalcin axis.

## Current cell and animal models for research, and translational challenges in the field

The continued investigation of bone mineralisation and energy metabolism regulation necessitates a concentrated effort from the scientific community. There is a timely need to adopt advanced cellular models, genetic animal models and pharmacological approaches to provide conclusive and cumulative evidence. At present, there is standard utilisation of immortalised single-cell type cell lines, for example, the murine pre-osteoblastic MC3T3 cells. Since there is evidence of bone paracrine and autocrine signalling which regulates the metabolic function and bone formation [43, 44, 45∗] and influences the energetic demands of the body, there is an inherent need to adopt 3D and co-culture systems to investigate the metabolic roles of bone. Recent evidence suggests an increased capacity of osteoblast to mineralise *in vitro* when cultured in such conditions, with an enhanced osteogenic and osteocytic capacity of primary cells [46, 47, 48]. The mimicking of bone tissue architecture by utilising 3D culture systems is an important step towards recreating the specific tissue microenvironments of the native tissue and is an important tool in assessing specific questions of cell biology.

In concert, the utilisation of advanced animal models is necessary to reflect the *in vivo* situation. Such work has relied upon the use of mouse Cre models to facilitate tissue/cell-specific manipulations of genes integral for mineralisation regulation and to assess the whole-body associated metabolic phenotypes [6]. However, several bone-targeted Cre models have revealed unanticipated skeletal phenotypes, which are likely due to recombinant Cre expression on non-bone cells [49]. One step towards reducing the off-target Cre expression is the utilisation of inducible mouse models, such as the tamoxifen-inducible Cre, to allow for temporal tissue-specific gene expression changes. However, this method is not without drawbacks, primarily that the dosing of inducer agents (e.g. tamoxifen) requires optimisation and can result in additional alterations to bone formation, resorption and function *in vivo* which may become contraindicating when interpreting overall metabolic phenotypes and regulation of glucose homeostasis in animal models [50]. Despite the limitations of Cre models, careful design and use of appropriate controls still provide a useful tool to assess mineralisation regulation and contribution to energy homeostasis *in vivo.*

Clinical science has also revealed interesting findings in the bone-energy metabolism field. Notably, this has occurred following the utilisation of anti-diabetic drugs which have been found to influence bone metabolism in neutral, positive, and negative manners. For example, thiazolidinediones, a class of medicine used to manage and treat T2DM, are associated with reduced BMD and elevated fracture risk, with comparable negative effects on bone mineralisation seen following rosiglitazone and pioglitozone administration [51]. It has been suggested that these drugs, utilised to improve glucose metabolism, resulting in loss of the inhibitory effect of peroxisome proliferator-activated receptor gamma (PPAR-ƴ) on osteoclast differentiation, increased sclerostin and Dickkopf WNT Signaling Pathway Inhibitor 1) (DKK1) expression in osteocytes, with an increased adipose volume in bone marrow [52]. This work indicates a link between pharmaceutical agents targeting bone disorders and systemic glucose homeostasis and illuminates the need for careful consideration of patient risks for T2DM development when determining appropriate care packages for bone mineralisation disorders.

To facilitate a translational output of the basic research in the emerging field of the bone-glucose axis, there is a need to adopt multiple and advanced basic research methodologies coupled with animal modelling. This research should be complemented with continued integration of clinical findings whereby current pharmacological practices for treating T2DM or bone mineralisation disorders must be assessed for confounding effects in either direction. Such work will continue to illuminate novel pathways mediating the bone-glucose axis and will aid in the development of personalised care, particularly if coupled with GWAS and large-scale data sets (e.g. phospho-proteomics and metabolomics) to identify key risk factors in both male and female patients such as specific genetic polymorphisms which may influence response to pharmacological agents.

## Conclusion

The last decade has revealed that bone acts well beyond its established role as a supporting framework for the body. Indeed, bone is exemplified as an endocrine organ with bone-derived hormones acting in a systemic manner to influence glucose metabolism. Regulators of bone, including both pro- and anti-mineralisation factors, are emerging as metabolic coordinators which are capable of influencing glucose metabolism and are associated with T2D risks. The continued investigation of bone-specific factors and regulators will enhance our understanding of cross-tissue glucose homeostasis and metabolic disorder risk, identifying novel targets for the prediction, prevention, and treatment of T2D and associated health complications.

## Funding

The authors are grateful to the 10.13039/501100000268Biotechnology and Biological Sciences Research Council (10.13039/501100000268BBSRC) for Institute Strategic Programme Grant Funding BB/J004316/1 to VEM. For the purpose of open access, the author has applied a Creative Commons Attribution CC BY public copyright license to any Author Accepted Manuscript version arising from this submission.

## Declaration of competing interest

The authors declare that they have no known competing financial interests or personal relationships that could have appeared to influence the work reported in this paper.

## Data Availability

No data was used for the research described in the article.

## References

[1] Qin L., Liu W., Cao H., Xiao G. (2020). Molecular mechanosensors in osteocytes. Bone Research.

[2] Suchacki K.J., Tavares A.A.S., Mattiucci D., Scheller E.L., Papanastasiou G., Gray C. (2020). Bone marrow adipose tissue is a unique adipose subtype with distinct roles in glucose homeostasis. Nat Commun.

[3] Wei J., Hanna T., Suda N., Karsenty G., Ducy P. (2014). Osteocalcin promotes β-cell proliferation during development and adulthood through Gprc6a. Diabetes.

[4] Bilinski W.J., Stefanska A., Szternel L., Bergmann K., Siodmiak J., Krintus M. (2022). Relationships between bone turnover markers and factors associated with metabolic syndrome in prepubertal girls and boys. Nutrients.

[5] Guo Y.-C., Yuan Q. (2015). Fibroblast growth factor 23 and bone mineralisation. Int J Oral Sci.

[bib6] Roberts F.L., Rashdan N.A., Phadwal K., Markby G.R., Dillon S., Zoll J. (2021). Osteoblast-specific deficiency of ectonucleotide pyrophosphatase or phosphodiesterase-1 engenders insulin resistance in high-fat diet fed mice. J Cell Physiol.

[7] Bhatti J.S., Bhatti G.K., Mastana S.S., Ralhan S., Joshi A., Tewari R. (2010). ENPP1/PC-1 K121Q polymorphism and genetic susceptibility to type 2 diabetes in North Indians. Mol Cell Biochem.

[8] Huesa C., Zhu D., Glover J.D., Ferron M., Karsenty G., Milne E.M. (2014). Deficiency of the bone mineralization inhibitor NPP1 protects mice against obesity and diabetes. Disease Models & Mechanisms.

[9] Flammier S., Peyruchaud O., Bourguillault F., Duboeuf F., Davignon J.L., Norman D.D. (2019). Osteoclast-derived autotaxin, a distinguishing factor for inflammatory bone loss. Arthritis Rheumatol.

[10] Dillon S., Staines K.A., Millán J.L., Farquharson C. (2019). How to build a bone: PHOSPHO1, biomineralization, and beyond. JBMR Plus.

[11] Suchacki K.J., Morton N.M., Vary C., Huesa C., Yadav M.C., Thomas B.J. (2020). PHOSPHO1 is a skeletal regulator of insulin resistance and obesity. BMC Biol.

[12] Jiang M., Chavarria T.E., Yuan B., Lodish H.F., Huang N.-J. (2020). Phosphocholine accumulation and PHOSPHO1 depletion promote adipose tissue thermogenesis. Proc Natl Acad Sci USA.

[13] Yadav M.C., Simão A.M.S., Narisawa S., Huesa C., McKee M.D., Farquharson C. (2011). Loss of skeletal mineralization by the simultaneous ablation of PHOSPHO1 and alkaline phosphatase function: a unified model of the mechanisms of initiation of skeletal calcification. J Bone Miner Res.

[14] Estève D., Galitzky J., Bouloumié A., Fonta C., Buchet R., Magne D. (2016). Multiple functions of MSCA-1/TNAP in adult mesenchymal progenitor/stromal cells. Stem Cell Int.

[bib15] Zhang Z., Nam H.K., Crouch S., Hatch N.E. (2021). Tissue nonspecific alkaline phosphatase (TNAP) function in bone and muscle progenitor cells: control of mitochondrial respiration and ATP production. Int J Mol Sci.

[16] Chirambo G., van Niekerk C., Crowther N.J. (2017). Specific knock-down of tissue non-specific alkaline phosphatase mRNA levels inhibits intracellular lipid accumulation in 3T3-L1 and HepG2 cells. Int J Exp Pathol.

[17] Liu W., Zhang L., Xuan K., Hu C., Liu S., Liao L. (2018). Alpl prevents bone ageing sensitivity by specifically regulating senescence and differentiation in mesenchymal stem cells. Bone Res.

[bib18] Sun Y., Rahbani J.F., Jedrychowski M.P., Riley C.L., Vidoni S., Bogoslavski D. (2021). Mitochondrial TNAP controls thermogenesis by hydrolysis of phosphocreatine. Nature.

[19] Ikeda K., Yamada T. (2020). UCP1 dependent and independent thermogenesis in Brown and beige adipocytes. Front Endocrinol.

[20] Boskey A.L., Maresca M., Ullrich W., Doty S.B., Butler W.T., Prince C.W. (1993). Osteopontin-hydroxyapatite interactions in vitro: inhibition of hydroxyapatite formation and growth in a gelatin-gel. Bone Miner.

[21] Daniele G., Guardado Mendoza R., Winnier D., Fiorentino T.V., Pengou Z., Cornell J. (2014). The inflammatory status score including IL-6, TNF-α, osteopontin, fractalkine, MCP-1 and adiponectin underlies whole-body insulin resistance and hyperglycemia in type 2 diabetes mellitus. Acta Diabetol.

[22] Papaioannou I., Pantazidou G., Kokkalis Z., Georgopoulos N., Jelastopulu E. (2021). Systematic review: are the elderly with diabetes mellitus type 2 prone to fragility fractures?. Cureus.

[23] Liu L.-F., Shen W.-J., Zhang Z.H., Wang L.J., Kraemer F.B. (2010). Adipocytes decrease Runx2 expression in osteoblastic cells: roles of PPARγ and adiponectin. J Cell Physiol.

[24] Kajimura D., Lee Ha W., Riley Kyle J., Arteaga-Solis E., Ferron M., Zhou B. (2013). Adiponectin regulates bone mass via opposite central and peripheral mechanisms through FoxO1. Cell Metabol.

[25] Tsuji K., Maeda T., Kawane T., Matsunuma A., Horiuchi N. (2010). Leptin stimulates fibroblast growth factor 23 expression in bone and suppresses renal 1alpha,25-dihydroxyvitamin D3 synthesis in leptin-deficient mice. J Bone Miner Res.

[26] Ducy P., Amling M., Takeda S., Priemel M., Schilling A.F., Beil F.T. (2000). Leptin inhibits bone formation through a hypothalamic relay: a central control of bone mass. Cell.

[27] Kawada T. (2022). The risk of hip fractures in patients with type 2 diabetes mellitus and prediabetes. Bone.

[28] Bonnet N. (2017). Bone-derived factors: a new gateway to regulate glycemia. Calcif Tissue Int.

[29] Rendina D., D'Elia L., Evangelista M., De Filippo G., Giaquinto A., Abate V. (2021). Metabolic syndrome is associated to an increased risk of low bone mineral density in free-living women with suspected osteoporosis. J Endocrinol Invest.

[30] Zhou R., Dang X., Sprague R.S., Mustafa S.J., Zhou Z. (2020). Alteration of purinergic signaling in diabetes: focus on vascular function. J Mol Cell Cardiol.

[31] Seref-Ferlengez Z., Maung S., Schaffler M.B., Spray D.C., Suadicani S.O., Thi M.M. (2016). P2X7R-Panx1 complex impairs bone mechanosignaling under high glucose levels associated with type-1 diabetes. PLoS One.

[32] Chen Y., Wu Y-y, Si H-b, Lu Y-r, Shen B. (2021). Mechanistic insights into AMPK-SIRT3 positive feedback loop-mediated chondrocyte mitochondrial quality control in osteoarthritis pathogenesis. Pharmacol Res.

[33] Malluche H.H., Davenport D.L., Cantor T., Monier-Faugere M.C. (2014). Bone mineral density and serum biochemical predictors of bone loss in patients with CKD on dialysis. Clin J Am Soc Nephrol.

[34] Karava V., Christoforidis A., Kondou A., Dotis J., Printza N. (2021). Update on the crosstalk between adipose tissue and mineral balance in general population and chronic kidney disease. Frontiers in Pediatrics.

[bib35] Green C.L., Pak H.H., Richardson N.E., Flores V., Yu D., Tomasiewicz J.L. (2022). Sex and genetic background define the metabolic, physiologic, and molecular response to protein restriction. Cell Metabol.

[36] Lee S.Y., Fam K.D., Chia K.L., Yap M.M.C., Goh J., Yeo K.P. (2020). Age-related bone loss is associated with FGF21 but not IGFBP1 in healthy adults. Exp Physiol.

[37] Dong K., Hao P., Xu S., Liu S., Zhou W., Yue X. (2017). Alpha-lipoic acid alleviates high-glucose suppressed osteogenic differentiation of MC3T3-E1 cells antioxidant effect and PI3K/akt signaling pathway. Cell Physiol Biochem.

[38] Medeiros C., Wallace J.M. (2022). High glucose-induced inhibition of osteoblast like MC3T3-E1 differentiation promotes mitochondrial perturbations. PLoS One.

[39] Karner C.M., Long F. (2018). Glucose metabolism in bone. Bone.

[40] Wang S., Deng Z., Ma Y., Jin J., Qi F., Li S. (2020). The role of autophagy and mitophagy in bone metabolic disorders. Int J Biol Sci.

[41] Ru J.Y., Wang Y.F. (2020). Osteocyte apoptosis: the roles and key molecular mechanisms in resorption-related bone diseases. Cell Death Dis.

[42] Rashdan N.A., Sim A.M., Cui L., Phadwal K., Roberts F.L., Carter R. (2020). Osteocalcin regulates arterial calcification via altered Wnt signaling and glucose metabolism. J Bone Miner Res.

[43] Lu Y., Yang Y., Xiao L., Li S., Liao X., Liu H. (2021). Autocrine and paracrine effects of vascular endothelial cells promote cutaneous wound healing. BioMed Res Int.

[44] Suzuki H., Ohshima N., Tatei K., Taniguchi T., Sato S., Izumi T. (2020). The role of autonomously secreted PGE2 and its autocrine/paracrine effect on bone matrix mineralization at the different stages of differentiating MC3T3-E1 cells. Biochem Biophys Res Commun.

[bib45] Ahmed A.S.I., Sheng M.H.C., Lau K.-H.W., Wilson S.M., Wongworawat M.D., Tang X. (2022). Calcium released by osteoclastic resorption stimulates autocrine/paracrine activities in local osteogenic cells to promote coupled bone formation. Am J Physiol Cell Physiol.

[46] Payr S., Rosado-Balmayor E., Tiefenboeck T., Schuseil T., Unger M., Seeliger C. (2021). Direct comparison of 3D and 2D cultivation reveals higher osteogenic capacity of elderly osteoblasts in 3D. J Orthop Surg Res.

[47] Bicer M., Cottrell G.S., Widera D. (2021). Impact of 3D cell culture on bone regeneration potential of mesenchymal stromal cells. Stem Cell Res Ther.

[48] Nasello G., Alamán-Díez P., Schiavi J., Pérez M., McNamara L., García-Aznar J.M. (2020). Primary human osteoblasts cultured in a 3D microenvironment create a unique representative model of their differentiation into osteocytes. Front Bioeng Biotechnol.

[49] Lim J., Burclaff J., He G., Mills J.C., Long F. (2017). Unintended targeting of Dmp1-Cre reveals a critical role for Bmpr1a signaling in the gastrointestinal mesenchyme of adult mice. Bone Res.

[50] Xie Z., McGrath C., Sankaran J., Styner M., Little-Letsinger S., Dudakovic A. (2021). Low-dose tamoxifen induces significant bone formation in mice. JBMR Plus.

[51] Zhu Z.-N., Jiang Y.-F., Ding T. (2014). Risk of fracture with thiazolidinediones: an updated meta-analysis of randomized clinical trials. Bone.

[52] Kheniser K.G., Polanco Santos C.M., Kashyap S.R. (2018). The effects of diabetes therapy on bone: a clinical perspective. J Diabetes Complicat.

